# Evaluating Aftereffects of Short-Duration Transcranial Random Noise Stimulation on Cortical Excitability

**DOI:** 10.1155/2011/105927

**Published:** 2011-07-26

**Authors:** Leila Chaieb, Walter Paulus, Andrea Antal

**Affiliations:** Department of Clinical Neurophysiology, Georg-August University, Robert Koch Stra*β*e 40, 37075 Göttingen, Germany

## Abstract

A 10-minute application of highfrequency (100–640 Hz) transcranial random noise stimulation (tRNS) over the primary motor cortex (M1) increases baseline levels of cortical excitability, lasting around 1 hr poststimulation Terney et al. (2008). We have extended previous work demonstrating this effect by decreasing the stimulation duration to 4, 5, and 6 minutes to assess whether a shorter duration of tRNS can also induce a change in cortical excitability. Single-pulse monophasic transcranial magnetic stimulation (TMS) was used to measure baseline levels of cortical excitability before and after tRNS. A 5- and 6-minute tRNS application induced a significant facilitation. 4-minute tRNS produced no significant aftereffects on corticospinal excitability. Plastic after effects after tRNS on corticospinal excitability require a minimal stimulation duration of 5 minutes. However, the duration of the aftereffect of 5-min tRNS is very short compared to previous studies using tRNS. Developing different transcranial stimulation techniques may be fundamental in understanding how excitatory and inhibitory networks in the human brain can be modulated and how each technique can be optimised for a controlled and effective application.

## 1. Introduction

Intermittent theta burst stimulation (iTBS) [1], high-frequency repetitive transcranial magnetic stimulation (rTMS) [2], anodal transcranial direct current stimulation (tDCS) [3], paired associative stimulation (PAS) [4], and now high-frequency random noise stimulation (tRNS) [5] are all techniques implemented in order to induce sustained elevations of cortical excitability, when applied over the primary motor cortex (M1). 

These methods of transiently modulating neuroplastic-like effects in the human cortex are generally short-lived, and dependent upon the stimulus duration and intensity. In the case of theta burst stimulation and anodal transcranial direct current stimulation, after effects are well characterised [3], and here we aim to evaluate the longer after effects observed poststimulation with high-frequency transcranial random noise (tRNS). 

The aim of this study was to investigate whether a threshold stimulation duration is necessary to produce sustained and measurable after effects on corticospinal excitability; a variable that has not been previously studied with regard to applications of tRNS. There is growing interest in understanding the effect of applying transcranial stimulation techniques in intervals of short-duration applications to optimise or prolong after effects; this has already been shown with tDCS [6]. A study published by our own group reports that both a 10-minute, 1 mA application of full spectrum (tRNS) or high-frequency transcranial random noise (HF tRNS) over the primary motor cortex (M1) can induce elevations in cortical excitability outlasting the duration of stimulation [5]. However, it is yet unknown exactly how long these observable after effects are able to endure, whether they are duration dependent, and if shorter stimulation durations are still able to modulate transcranial magnetic stimulation (TMS-) induced motor-evoked potentials (MEPs), a global measure of corticospinal excitability [7]. Only two previous studies have been published regarding the effects of tRNS over the M1; both of these used varying stimulation durations and methodological approaches. The authors observed an attenuation in the BOLD response during the performance of a simple finger-tapping task after a 4 min stimulation duration [8] and an increase in corticospinal excitability using TMS measures after a 10-min stimulation [5]. Our aims were to establish whether short-duration stimulation applications were able to induce measurable after effects, and if so, how long they were able to endure poststimulation; a stimulation variable that has not yet been investigated for the application of tRNS. 4, 5, and 6 mins tRN stimulation durations were selected to try to identify the threshold at which cortical excitability enhancements could be observed. These stimulation durations were chosen as previous studies report that cortical facilitation could be observed after 10 mins tRNS [5] and an attenuation of the BOLD response after 4 mins tRN stimulation [8]. We postulated that the threshold for excitability would lie between these two stimulation durations. Here we would like to briefly communicate how we have assessed the after effects of short-duration stimulation applications of the reported application of tRNS.

## 2. Materials and Methods

### 2.1. Subjects

Twenty-two healthy subjects (18 male, age ranges: 20–30 years); 15 right handed according to the Edinburgh Handedness Inventory [9] participated in the study. All participants were informed of all aspects of the experiments and gave written consent. None of the participants, suffered from any neurological or psychological disorders nor had any metal implants or implanted devices, took any relevant medication regularly or prior to their participation. All aspects of the protocol conformed to the Declaration of Helsinki and were approved by the Ethics Committee of the University of Göttingen.

### 2.2. Transcranial Random Noise Stimulation of Motor Cortex

tRNS was delivered by a battery-driven electrical stimulator (Version DC-Stimulator-Plus, NeuroConn GmbH, Ilmenau, Germany) through conductive rubber electrodes, placed in two saline-soaked sponges. In the stimulation mode “noise”, there is a random level of current generated for every sample (sampling rate 1280 sps). The random numbers are normally distributed; the probability density function follows a bell-shaped curve. In the frequency spectrum, all coefficients have a similar size (“white noise”). The noise signal contains all frequencies up to half of the sampling rate, that is, a maximum of 640 Hz (101 Hz–640 Hz). Due to the statistical characteristics, the signal has no DC offset, provided that the offset is set to zero.

The stimulation electrode was placed over the left motor cortex, which was determined prior to stimulation by single-pulse TMS. The reference electrode was placed over the contralateral orbit. The size of the stimulation electrode was 4 × 4 cm, and the reference electrode was 6 × 14 cm and was fixated to the head by elastic bands. High-frequency tRNS was applied for 4, 5, and 6 minutes with a current strength of 1000 *μ*A. The maximal current density was 62.5 *μ*A/cm^2^ over the motor cortex, which is below the safety parameters accepted for tDCS [10]. The current density was 12 *μ*A/cm^2^ at the electrode placed over the contralateral orbit. For sham stimulation, the current was turned up for 30 sec at the beginning of the stimulation, and then subsequently turned off. However, the screen on the stimulator did show the remaining time until the end of the stimulation, as in the verum stimulation condition. Subjects were blinded as to active and sham stimulation conditions.

### 2.3. Measuring Corticospinal Excitability

To detect current-driven changes of excitability, motor-evoked potentials (MEPs) of the right first dorsal interosseus muscle (FDI) were recorded following stimulation of its motor-cortical representation field by single-pulse TMS. These were induced using a Magstim 200 magnetic stimulator (Magstim Company, Whiteland, Wales, UK), with a figure-of-eight standard double magnetic coil (diameter of one winding, 70 mm; peak magnetic field, 2.2 T; average inductance, 16.35 *μ*H). Surface electromyogram (EMG) was recorded from the right FDI through a pair of Ag-AgCl surface electrodes in a belly-tendon montage. Raw signals were amplified, band-pass filtered (2 Hz-3 kHz; sampling rate, 5 kHz), digitized with a micro 1401 AD converter (Cambridge Electronic Design, Cambridge, UK) controlled by Signal Software (Cambridge Electronic Design, version 2.13), and stored on a personal computer for offline analysis. Complete relaxation was controlled through auditory and visual feedback of EMG activity, and whenever it was necessary, the subject was instructed to relax. The coil was held tangentially to the skull, with the handle pointing backwards and laterally at 45° from the midline, resulting in a posterior-anterior direction of current flow in the brain. This orientation of the induced electrical field is thought to be optimal for predominantly transsynaptic mode of activation of pyramidal tract neurons synapsing onto the corticospinal system. The optimum position was defined as the site where TMS resulted consistently in the largest MEP in the resting muscle. The site was marked with a skin marker to ensure that the coil was held in the correct position throughout the experiment.

### 2.4. Experimental Procedure

Subjects were seated in a comfortable reclining chair with a mounted headrest throughout the experiments. Both experimental conditions were conducted by the same investigator, who was not blinded with regard to the stimulation session.

**4, 5, and 6 Minutes Full Spectrum tRNS**

Ten (4 min) and twelve (5- and 6-min) subjects participated in two experimental sessions, on separate days, at least 3 days apart to avoid carryover effects. The same subject group participated in the 5- and 6-min stimulation experiments, whilst there was an overlap between the subjects in the group used for the 4-min experimental session. The subjects received tRN and sham stimulation in a randomised and counterbalanced order. Resting motor threshold (RMT), active motor threshold (AMT), the intensity to evoke MEP of *∼*1 mV peak-to-peak amplitude (SI1 mV), and a baseline of TMS-evoked MEPs (40 stimuli) were recorded at 0.25 Hz prior to stimulation. Stimulus intensities (in percentage of maximal stimulator output) of TMS were determined at the beginning of each experiment. RMT was defined as the minimal output of the stimulator that induced a reliable MEP (*∼*50 *μ*V in amplitude) in at least three of six consecutive trials when the FDI muscle was completely relaxed. AMT was defined as the lowest stimulus intensity at which three of six consecutive stimuli elicited reliable MEP (*∼*200 *μ*V in amplitude) in the tonically contracting FDI muscle [7]. Immediately following stimulation, 40 single test-pulse MEPs were recorded using monophasic single-pulse TMS at 0.25 Hz, at 0-min, 5-min, 10 -min poststimulation and then every 10 minutes up to 60 min.

### 2.5. Calculations and Statistics

**Figure 1:**
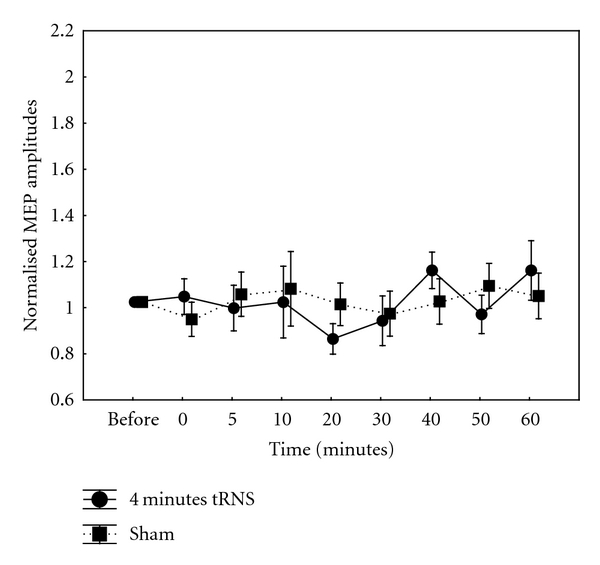
Normalised MEP amplitudes after a 4-minute, 1 mA application of full-spectrum tRNS over the human primary motor cortex. There was no significant increase in MEP amplitudes after a moderately short-duration tRNS application. Data are mean peak-to-peak MEP amplitudes over time points. Error bars indicate SEM.

**Figure 2:**
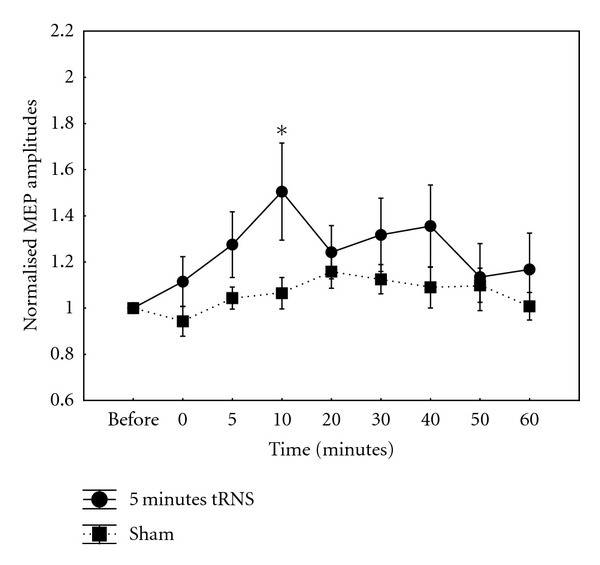
Normalised MEP amplitudes after a 5-minute, 1 mA application of full-spectrum tRNS over the human primary motor cortex. There was a significant increase in MEP amplitudes after a short-duration tRNS application at 10 mins poststimulation. Data are mean peak-to-peak MEP amplitudes over time points. Error bars indicate SEM. Asterisks denote significant time points (*P* < 0.05).

**Figure 3:**
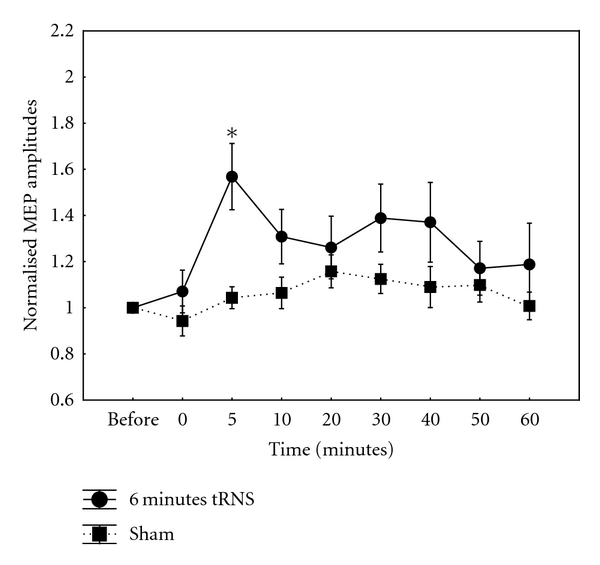
Normalised MEP amplitudes after a 6-minute, 1 mA application of full-spectrum tRNS over the human primary motor cortex. There was a significant increase in MEP amplitudes after a short-duration tRNS application at 5 mins poststimulation. Data are mean peak-to-peak MEP amplitudes over time points. Error bars indicate SEM. Asterisks denote significant time points (*P* < 0.05).

MEP amplitude (peak-to-peak SI1 mV) was automatically calculated by the NuCursor programme (IoN, UCL, London, UK), and the mean value was determined for each time point after data had been visually inspected offline, and any MEPs with EMG artefacts were rejected. No more than 10 EMG traces were rejected for each time point poststimulation, per experimental session. Repeated measures ANOVAs (CONDITION (4 or 5, 6 min tRNS versus sham) × TIME (before, 0, 5, 10, 20, 30, 40, 50, 60 mins poststimulation) were used to compare the different stimulation conditions. Effects were considered significant if *P* < 0.05; Bonferroni test was used to see significant differences between different stimulation condition at a given time point. Student's *t*-test was used to compare the baseline MEPs among stimulation conditions. All data are given as means + SEM. MEP amplitudes were normalised to baseline; all Figures 1–3 show normalised MEP amplitude values. Nonsphericity was checked and corrected for using a Greenhouse-Geisser analysis.

## 3. Results

All of the subjects tolerated the stimulation; none of the experimental sessions were interrupted due to side effects of the stimulation. The subjects reported no side effects after the stimulation.

**4 Minutes tRNS**

4-min tRNS did not induce an excitability increase, as previously observed after 10-min tRNS. Indeed, after 4-min tRNS, there was a tendency toward inhibition when compared to sham stimulation; however, this short-lived attenuation of cortical excitability was not significant. Repeated measures ANOVA revealed no significant effect of CONDITION (*F*(1,9) = 0.4, *P* = 0.54) and TIME (*F*(8,72) = 0.97, *P* = 0.5) (Figure 1). The interaction between CONDITION and TIME was also not significant (*F*(8,72) = 1.2, *P* = 0.31).

**5 Minutes tRNS**

5 min tRNS induced an excitability increase, as previously observed after 10-min tRNS [5]; however, the increase was significant at 10-min poststimulation (*t* = 2.31, *P* = 0.04). Repeated measures ANOVA revealed significant effect of CONDITION (*F*(1,11) = 7.38; *P* = 0.02) but not TIME (*F*(8,88) = 1.97, *P* = 0.06) (Figure 2). The interaction between CONDITION and TIME was also not significant (*F*(8,88) = 1.57, *P* = 0.15).

**6 Minutes tRNS**

6-min tRNS induced a stronger excitability increase than 5-min tRNS. The increase of MEP amplitude was significant at 5- (*t* = 3.88; *P* = 0.002), 10 min (*t* = 2.6, *P* = 0.03), and 30-min poststimulation (*t* = 2.8, *P* = 0.02). Repeated measures ANOVA revealed significant effect of CONDITION (*F*(1,11) = 10.34; *P* = 0.0008) and TIME (*F*(8,88) = 3.03, *P* = 0.004) (Figure 3). The interaction between CONDITION and TIME was also not significant (*F*(8,88) = 1.7, *P* = 0.11).

RMT, AMT, and SI1 mV baseline values were compared between tRNS and sham conditions using Student's *t*-test. There was no significant difference between tRNS and sham stimulation in any of the measurements (*P* > 0.4). Due to extensive overlap between subject groups and that some subjects did not participate in all stimulation conditions, an appropriate statistical analysis of baseline MEP amplitudes could not be implemented. Means and SDs of baseline values of each stimulation condition are provided here: 4 mins: mean: 1.04 mV, +/− 0.14; 5 mins: mean: 1.05 mV, +/− 0.13; 6 mins: mean: 1.05 mV, +/− 0.10; SHAM : mean: 0.95, +/− 0.14. Greenhouse-Geisser correction for nonsphericity for 5 and 6 mins stimulation duration was performed: 5 mins: CONDITION × TIME (*P* = 0.206); 6 mins: CONDITION × TIME (*P* = 0.169).

## 4. Discussion

The primary aim of this study was to investigate the stimulation duration threshold at which sustainable neuroplastic effects can be measured, using shorter stimulation applications of full spectrum tRNS, which enabled us to compare the inducible after effects of the initial study reporting the facilitatory effects on MEPs of 10 mins full-spectrum tRNS [5]. Full-spectrum tRNS was applied in order to compare MEP data with findings from another study reporting an attenuation of the BOLD response after a 4-min application of tRNS [8]. We can report that there appears to be a tRNS stimulation duration threshold for the induction of prolonged and measurable corticospinal after effects, when applied over the M1. Here we have demonstrated that after 5 mins tRNS, a significant elevation in MEP amplitudes was observed at 10 mins poststimulation. After 6 mins tRNS, a clear trend toward facilitation was observed at 5–10 mins and a nonsignificant increase until 30 mins poststimulation. Interestingly, a 4-minute tRNS application, also over the M1, did not induce an increase in corticospinal excitability; however, we have reported a blood oxygenated level-dependent change after 4-min tRNS in a previous study [8]. This may be attributed to differences within the subject populations and also the different evaluation methods used, as we did not until now measure the effects of 4 mins tRNS on corticospinal excitability. These techniques and their methodological approaches differ in a number of important respects: the number of participants in the neuroimaging study was significantly less [8] than that of the current study, and the inclusion of female participants within the participant group may have also contributed to the variation in response with regard to this TMS study, due to gender influences [11, 12]. A fundamental difference between the fMRI investigation was that participants were required to perform a finger-tapping task to be executed prior to and poststimulation, causing an activation of motor areas prior to tRNS stimulation—a major contrast to the tRNS protocol in the current investigation—which is while subjects are required to be passive and continually relaxed throughout the experimental sessions. It must also be noted that there is some intraindividual variability between the responses of the participants to the 4, 5, and 6 mins tRNS stimulation durations. However, the net effect of all responders was an increase in corticospinal excitability after 5 and 6 mins tRNS stimulation was applied. The observed increases in MEP amplitudes after 5 mins tRNS at 10-mins and after 6 mins tRNS at 5 mins poststimulation may be attributed to the increase in stimulation duration; we observed the same excitability enhancement early poststimulation in the initial study reporting the effects of tRNS on the M1 after a 10 min stimulation duration [5]. The slightly longer stimulation duration may be the reason why we observe faster induction of excitability enhancements at 5 mins (after 6 mins tRNS) instead of at 10 mins as in the case of 5 mins tRNS. A similar effect was reported in a seminal study looking at tDCS where the authors showed that increases in MEP amplitudes could be seen early poststimulation after an application of 13 mins anodal tDCS, but not the same early increase in MEP amplitude after 11 mins anodal tDCS, at 5 mins poststimulation [3]. Regarding the response of targeted neuronal populations, it may be that a larger subpopulation of neurons is being stimulated during the application of longer stimulation durations and that an increase in stimulation duration generates longer and more measurable after effects. As tRNS is an oscillatory current, we can only postulate that an increase in the number of neurons that are being stimulated contributes to the net effect of sustained cortical excitability and not to their geometry and orientation relative to the stimulating electrode as in the case of tDCS [3]. The observed after effects of high-frequency tRNS in the present study are similar to those reported after transcranial stimulation with anodal tDCS [3]. Anodal tDCS however, has one constraint: it is polarity dependent, a factor that does not govern cortical stimulation with tRNS. However, the intensities used to induce aftereffects and standard delivery of the stimulation are very similar; even the duration of stimulation in relation to observed aftereffects are not so far apart between these two approaches [5, 13]. 

 tRNS induced plasticity may be modulated by the continual activation and rectification of voltage-gated sodium channels [14]. Evidence from cultured rat hippocampal neurons suggests that activated inward sodium currents can give rise to weak depolarisations of the cell membrane; the same nonlinearity observed after repetitive high-frequency stimulation, and activation of sodium channels, in hippocampal cells, mirror the waveform of the tRNS effect and could account for the sustained excitability-enhancing effects of the stimulation [15]. 

In summary, a relatively short-duration (5- and 6-min) tRNS showed a trend toward facilitation; however, this effect was not as marked as those effects elicited after 10-min tRNS. Contrary to this effect, a 4-min tRNS application did not induce an excitability increase. These results suggest that a minimal stimulation duration is critical when aiming to modulate tRNS-induced after effects. This is also evident in earlier studies examining the inducible after effects of tDCS. Nitsche and Paulus [3] reported that a 13-minute anodal tDCS application was necessary to produce a sustained excitability increase of 9 minutes poststimulation. In contrast, 9-minute cathodal tDCS induced an excitability diminution lasting up to 60 minutes poststimulation [3]. We can see that although tRNS is a novel method of inducing sustained elevations of cortical excitability, it's after effects are comparable with those of more well-established transcranial stimulation techniques.

##  Conflict of Interests 

The authors would like to state that they have no conflict of interests.
